# Enhancing the Corrosion Resistance Performance of Mg-1.8Zn-1.74Gd-0.5Y-0.4Zr Biomaterial via Solution Treatment Process

**DOI:** 10.3390/ma13040836

**Published:** 2020-02-12

**Authors:** Ya Liu, Jiuba Wen, Huai Yao, Junguang He, Huan Li

**Affiliations:** 1 School of Materials Science and Engineering, Henan University of Science and Technology, Luoyang 471023, China; liuya_021@163.com (Y.L.); yaohuai@163.com (H.Y.); he.ellen@163.com (J.H.); hnlihuan@126.com (H.L.); 2 Collaborative Innovation Center of Nonferrous Metals of Henan Province, Luoyang 471023, China

**Keywords:** Mg-Zn-Zr-Gd-Y, solution treatment, microstructure, corrosion

## Abstract

Microstructure and corrosion behavior of the solution-treated Mg-1.8Zn-1.74Gd-0.5Y-0.4Zr (wt%) alloy were studied. The results of microstructure indicated that the second phases of as-cast alloy was mainly comprised of Mg_12_Zn(Gd,Y) phase, Mg_3_Zn_3_(Gd,Y)_2_ phase and (Mg,Zn)_3_(Gd,Y) phase. After solution treatment process, the second phase gradually dissolved into the matrix, and the grain size increased. The effect of microgalvanic corrosion between α-Mg matrix and second phase was also improved. At the range of 470~510 °C solution treatment temperature, the corrosion resistance of the samples increases at first and then decreases slightly at 510 °C. All the solution-treated Mg-Zn-Gd-Y-Zr samples exhibit better corrosion resistance in comparison with as-cast sample. The existence form of the remaining phase affects the morphology of the corroded surface that relatively complete dissolution with homogeneous microstructure makes the sample more effective to obtain uniform corrosion form. The optimum temperature for solution treatment is 490 °C, which shows a much better corrosion resistance and uniform corrosion form after soaking for a long time.

## 1. Introduction

Compared with traditional materials, magnesium (Mg) alloys, as biomedical materials, have a good application prospect due to the low elastic modulus (close to human bone), light quality, biodegradability and favorable biocompatibility [1,2,3]. In addition, the release of Mg^2+^ can provide the feasibility of better physiological repair [4]. Mg shows a significant tendency to corrode due to the low electrochemical potential, especially in chlorine containing solutions, including human body fluids or blood plasma [5]. Moreover, Mg alloys are vulnerable to microgalvanic corrosion. The excessive corrosion rate of magnesium alloy causes it to lose its mechanical integrity prematurely before tissues healing.

To overcome the rapid corrosion rate in the service period, a lot of studies have been done to improve the performance of corrosion, such as micro-alloying [6,7,8], surface modification and composites [9,10]. Alloying is one of the most effective methods. It should be noted that the first consideration in the selection of alloy elements is biocompatible [11]. The Mg-Zn-Zr alloys have been studied with excellent biocompatible [12]. Gadolinium (Gd), as rare earth element, is suitable for biomedical material [11,13]. The addition of Gd element can markedly ameliorate both the corrosion resistance and mechanical properties [7]. When the ratio of Zn/Gd is high, Mg_3_Zn_6_Gd phase, cubic Mg_2_Zn_3_Gd_2_ phase and binary Mg-Zn phase are the mainly phases in Mg_95.9_Zn_3.5_Gd_0.6_ (at%) alloy [14]. With the decrease of Zn/Gd ratio, Mg_3_Gd particles appear and the volume fraction of the Mg_3_Zn_3_Gd_2_ phase increases with the increasing of Gd in Mg-4.5Zn-xGd (x = 0, 2, 3 and 5) alloy [15]. Yttrium (Y) is widely used in Mg alloys due to the same crystal structure and standard electrode potential (−2.372 V) as those of Mg [16,17]. Jafari et al. [8] showed that Y in the matrix of as-cast Mg-5Zn can effectively slow the propagation of corrosion.

However, the large size phases in the matrix promote the local dissolution of the film layers reducing the corrosion resistance of the alloy [4]. The existence of a large number of second phases, in the as-cast alloy, accelerate the microgalvanic corrosion and produce pitting corrosion which is not conducive to the long-term service of the Mg alloy. Thus, heat treatment is utilized to coordinate the distribution of second phases to change the defects of Mg alloys [18,19,20,21]. As the solid solubility of Gd in Mg at 548 °C is about 4.53 at% (23.49 wt%), the use of Gd is more frequent [20]. Compared with many other elements, Zn and Y provide stronger solution strengthening effect [22]. Solution temperature and time are important factors affecting solution effect on properties and microstructure. It has been reported that the Mg-4.58Zn-2.6Gd-0.18Zr alloy, after solution treatment, the interdendritic eutectic structure was dissolved [23]. Zhang et al. [24] studied the effects of solution treatment at different temperatures. The results showed that the corrosion form of Mg-5Gd-1Zn-0.6Zr alloy solution treated at 400 °C for 5 h is more uniform and the corrosion rate is lower. By studying the holding time of solution treatment, Jafari et al. [19] showed that the corrosion rate in solution treated Mg-5Zn-1.5Y was decreased by a more homogenous distribution of Y with the prolong of time. The disadvantage is that grain coarsening after long-time solution treatment reduces mechanical properties with the extension of holding time. Meanwhile, Sara et al. [25] and Alvarez-Lopez et al. [26] presented that the decrease of grain size facilitated the corrosion rate reduction.

Considering the above reasons, the as-cast Mg-1.8Zn-1.74Gd-0.5Y-0.4Zr alloy was prepared followed with solution treatment. In the present paper, the suitable solution treatment temperature of the alloy is investigated and the solution treatment effect on the microstructure and corrosion resistance is conducted to discuss.

## 2. Experimental Details

The Mg-1.8Zn-1.74Gd-0.5Y-0.4Zr (wt%) alloy was prepared in an electrical induction furnace in the protective gas mixture of CO_2_ and SF_6_ (99:1 in volume ratio). The alloy was melted at 730 °C ensured all the necessary alloying elements entirely dissolved. Afterwards, the ingots were solution-treated at 470 °C, 490 °C and 510 °C for 8 h, and then quenched into water with a temperature of 65 °C (named as T4-470, T4-490 and T4-510, respectively).

All the specimens, for the microstructure observations, were ground, polished and cleaned. The microstructure was investigated by optical microscopy (OM, OLYMPUS PMG3, Tokyo, Japan), scanning electronic microscopy (SEM, JSM-5610LV, JEOL, Tokyo, Japan equipped with energy dispersive spectroscopy (EDS)). Phases of specimens were measured using transmission electron microscopy (TEM, JEM-2010, JEOL, Tokyo, Japan) and X-ray diffraction (XRD, D8 ADVANCE, Bruker, Karlsruhe, Germany). The grain size was measured with Nano Measurer software (Shanghai, China). ImageJ software (National Institutes of Health (NIH), Bethesda, MD, USA) was used to quantify the volume fraction of second phase.

The electrochemical tests of the samples were evaluated using Autolab PGSTAT128N (Metrohm, Herisau, Switzerland) electrochemical workstation with an exposed area of 1 cm^2^. The open circuit potential (OCP) test started immediately after the samples exposed to simulated body fluid (SBF) (8.0 g/L NaCl, 1.0 g/L glucose, 0.4 g/L KCl, 0.35 g/L NaHCO_3_, 0.14 g/L CaCl_2_, 0.1 g/L MgCl_2_·6H_2_O, 0.06 g/L Na_2_HPO_4_·12H_2_O, 0.06 g/L KH_2_PO_4_, and 0.06 g/L MgSO_4_·7H_2_O) [7] and went on for 3600 s at 37 °C. After 3600 s stabilization, the polarization curve was measured from −250 mV to +400 mV relative to the value of OCP vs. SEC at a scanning rate of 1 mV/s. The self-corrosion potential (*E_corr_*) and corrosion current density (*I_corr_*) were calculated by Tafel extrapolation. The corresponding corrosion rate (*P_i_*) was calculated using Equation (1) [2]

*P_i_* = 22.85*I_corr_* (1)

The immersing specimens for weight loss test were Φ 18 mm × 3 mm. To keep the corrosion environment relatively stable, SBF was replaced every 24 h in a thermostat water bath at 37 ± 0.1 °C. For each test material, an average of three groups of samples were carried out. The corrosion rate (mm/year) of weight loss test was calculated by Equation (2) [4]

(2) Corrosion Rate=K×W/A×ρ×t

where *K* = 87.6, *W* is the mass loss (mg), *A* is the surface area of the specimen exposed to solution (cm^2^), ρ is the sample density (g/cm^3^) and t is the immersion time (h).

After immersion, samples were ultrasonically cleaned in a boiling chromic acid solution to remove the corrosion products. Afterwards, surface morphologies of the cleaned sample were analyzed by using confocal laser scanning microscopy (CLSM, OLYMPUS LEXT OLS4000, Tokyo, Japan).

## 3. Results and Discussion

### 3.1. Microstructures Analysis

**Figure 1:**
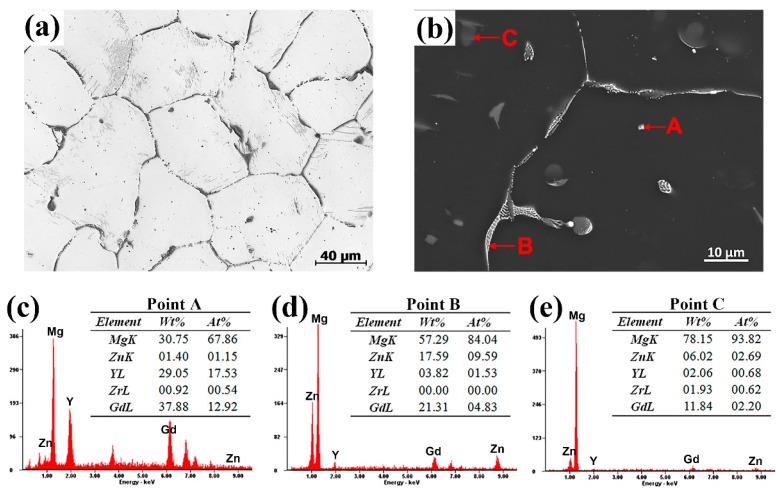
Optical microscopy (OM) and scanning electronic microscopy (SEM) images of the as-cast sample. (**a**) OM image; (**b**) SEM image; (**c**) energy dispersive spectroscopy (EDS) result at the point A; (**d**) EDS result at the point B; (**e**) EDS result at the point C.

Typical microstructures and corresponding EDS results of the as-cast sample are shown in Figure 1. From OM image (Figure 1a), it can be seen that the as-cast alloy has a nearly equiaxed dendrite α-Mg grain structure. It is mainly composed of α-Mg matrix, lamellar structure, cuboid-like phase and skeleton-like eutectic phase. In Figure 1c–e, the semi-quantitative EDS results indicate that the cuboid-like phase, skeleton-like phase and lamellar structure (points A, B and C in Figure 1b) have approximate stoichiometric composition of (Mg,Zn)_3_(Gd,Y), Mg_3_Zn_3_(Gd,Y)_2_ and Mg_12_Zn(Gd,Y), respectively [27]. When the ratio value is close to 1.5, the skeleton-like phase at point B is Mg_3_Zn_3_(Gd,Y)_2_. While the lamellar structure is Mg_12_Zn(Gd,Y) phase as the ratio value is 0.93 at point C in Figure 1b.

**Figure 2:**
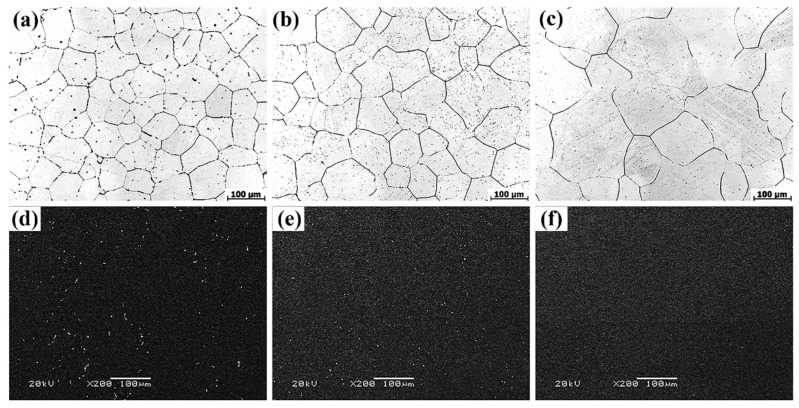
OM (**a**–**c**) and SEM (**d**–**f**) images of the solution-treated samples: (**a**,**d**) T4-470 sample; (**b**,**e**) T4-490 sample; (**c**,**f**) T4-510 sample.

**Figure 3:**
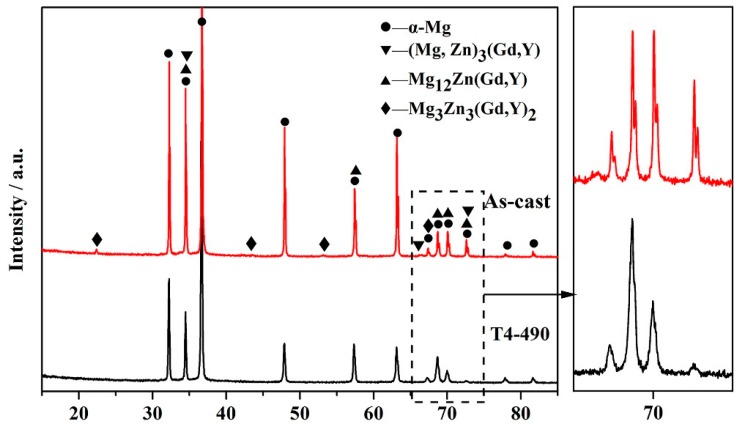
XRD patterns of the as-cast and T4-490 samples.

The microstructures of solution-treated samples are depicted in Figure 2. Figure 2a–c are the OM images of T4-470, T4-490 and T4-510, respectively. The grain size increased rapidly with the increasing of temperature. When the temperature was 470 °C, 490 °C and 510 °C, the average grain size was about 79.0 μm, 95.1 μm and 112.5 μm, respectively. After solution treatment from 470 °C to 490 °C, the grain boundaries approached straightness. When the solution temperature rose to 510 °C, the grain size was anomalous with a noticeable coarsening tendency due to the grain mergence [21]. SEM results from Figure 2d–e reveals that a mass of the second phases vanished at 470 °C, only locally residual second phases were observed. The proportion of second phases decreased with the increasing of solution temperature. The Mg_12_Zn(Gd,Y) phase completely dissolved into the matrix with a small amount of residual similar ellipse phase left. As it is evident, the majority of second phases were dissolved into the matrix at 490 °C with more evenly distributed remaining second phases (Figure 2d). The α-Mg matrix and trace residual second phases were considered as the main constitution of the T4-490 and T4-510 samples. It can be seen from Table 1 that the volume fraction of the second phase after solution at 490 °C and 510 °C was 0.34% and 0.11%, respectively. Figure 3 displays the XRD patterns of the as-cast and T4-490 samples. The as-cast sample is dominantly made up of α-Mg, Mg_12_Zn(Gd,Y) phase, Mg_3_Zn_3_(Gd,Y)_2_ phase and (Mg,Zn)_3_(Gd,Y) phase. It has also been previously reported in the Mg–Zn–Zr–Y, Mg–Zn–Zr–Gd, and Mg–Zn–Zr–Y–Gd series alloys [21,22,23,28]. For the T4-490 sample, the specimen only consisted of the α-Mg, which means that a slew of second phases dissolved into the matrix and the trace amount of second phases may not have been detected.

**Figure 4:**
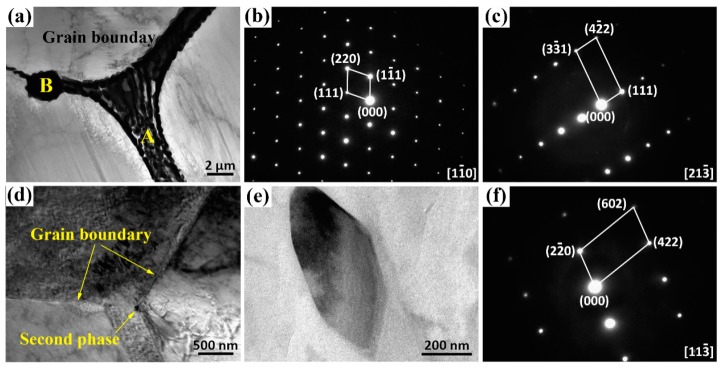
TEM images of the as-cast (**a**–**c**) and T4-490 (**b**–**f**) samples: (**a**) BF TEM image for as-cast sample; (**b**) selected area electron diffraction (SAED) patterns of point A in (a); (**c**) SAED patterns of point B in (a); (**d**,**e**) BF TEM images for T4-490 sample; (**f**) SAED pattern of the second phase in (**e**).

The TEM analysis was used to further confirm the microstructure compositions, as shown in Figure 4. The selected area electron diffraction (SAED) of skeleton-like phase presented in Figure 4b was found to have a face-centered cubic (FCC) structure with a lattice constant of a = 0.703 nm similar to the Mg_3_Zn_3_Y_2_ phase (0.683 nm). Hence, the skeleton-like phase at the grain boundaries was affirmed as Mg_3_Zn_3_(Gd,Y)_2_ type phase. Meanwhile, it can be confirmed that the cuboid-like phase in Figure 4a was (Mg,Zn)_3_(Gd,Y) phase through SAED patterns (Figure 4c) and our previous study [28]. After solution treatment at 490 °C, the undissolved phase existed at the vaguely visible and straight grain boundary (Figure 4d). Figure 4e reveals the undissolved phase at 490 °C, corresponding to the Mg_3_Zn_3_(Gd,Y)_2_ phase through SAED patterns (Figure 4f) was consistent with the previous studies [22].

### 3.2. Electrochemical Tests and Immersion Tests

**Figure 5:**
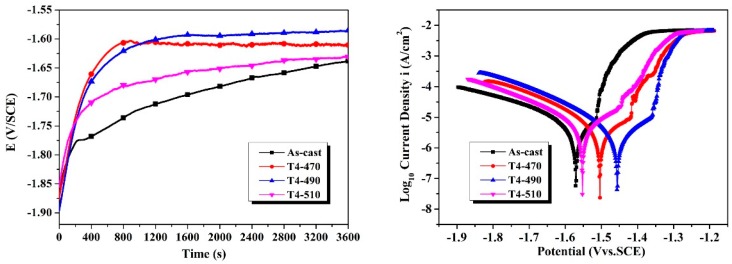
Electrochemical tests: (**a**) open circuit potential (OCP) curves; (**b**) potentiodynamic polarization curves after 3600 s stabilization.

Figure 5a presents the variation of the OCP as a function of time measured from the samples immersed in SBF for up to 3600 s. Initially, the OCP values of the samples were close to −1.896 V to −1.855 V. In the range of potential, the OCP curves illustrated a similar trend: the potential of the samples gradually moved to a nobler direction, and tended to be constant at a later stage of immersion indicating the formation of a protective surface film [29,30]. It can be seen that the OCP of the solution-treated samples was significant towards to more positive direction than that of the as-cast sample. Since the potential of the three types second phase in the alloy is higher than that of the α-Mg matrix [28], the dissolution of the second phase increased the potential of the matrix during the solid solution process. The T4-490 sample stabilized at −1.586 V nobler than the other samples. The steady OCP value of the T4-490 sample was about 52, 24, 45 mV higher than that of as-cast, T4-470, T4-510 samples, respectively. Figure 5b illustrates the polarization curves of the samples tested in SBF after 3600 s stabilization. The similarity of the curves indicates that the samples showed a similar electrochemical corrosion mechanism. The fitting results obtained from polarization curves are listed in Table 2. It is shown that the as-cast sample exhibits a more negative self-corrosion potential (*E_corr_*) at −1.571 V. The solution treated samples shifted to nobler direction compared with that of the as-cast sample. The corrosion current density (*I_corr_*) values of the samples under different conditions presented the following trend: T4-49510 > as-cast > T4-470 > T4-490. The lower value of *I_corr_* means the better corrosion resistance. In addition, a significant breakdown potential (*E_b_*) occurred in the anode region of all the polarization curves. The *E_b_* value for as-cast sample was more negative, which means the localized corrosion was likely to occur on as-cast sample.

**Figure 6:**
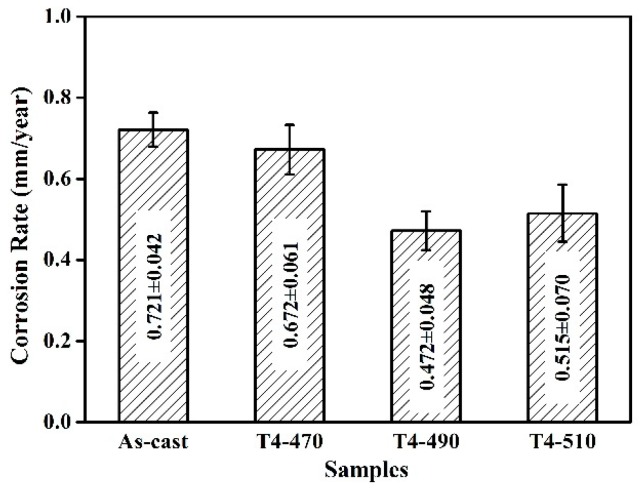
Corrosion rate of the samples immersing in simulated body fluid (SBF) at 37 °C for 120 h.

The corrosion rates of the samples calculated by weight loss test after immersing in SBF at 37 ± 1 °C for 120 h are presented in Figure 6. The corrosion rate of the samples decreased firstly and then increased lightly similar to the trend of electrochemical test. Noticeably, the corrosion rate of the as-cast sample had the highest value (0.721 ± 0.042 mm/year) among the samples. The main reason for the high corrosion rate is the large volume fraction of the second phase. In the course of corrosion, the Mg matrix acted as the anode phase because of the lower potential in comparison with the second phase leading to the microgalvanic corrosion. After solution treatment, the corrosion rate of T4-470 decreased slightly due to the partly dissolved second phase. The partial dissolution of the second phase reduced the occurrence of microgalvanic corrosion, and also improved the value of potential. The lowest corrosion rate was obtained at 490 °C with a value of 0.472 ± 0.048 mm/year. At this temperature, the residual second phases volume fraction was only 0.34% ± 0.13% with a relatively homogeneous dissolution. After solution treatment, the remaining phase with small size played a significant role in reducing the microgalvanic corrosion [4,31]. However, the T4-510 sample had a higher corrosion rate, indicating that the second phase was not the only factor affecting corrosion. In the current research, on the one hand, the decreased volume fraction of second phase after solution treatment may have increased the potential of matrix and weaken the microgalvanic corrosion. Thus, to some extent, the reduction of the second phase was in a favorable position for corrosion resistance. On the other hand, with the increase of solution treatment temperature, the grains grew obviously, which played a negative role on the corrosion resistance [32]. Thus, when the grain growth was not obvious, the control of corrosion resistance was dominated by the reduction of the second phase. The corrosion rate was correspondingly decreased. When the solution treatment temperature increased and the grain growth was obvious, namely 510 °C, the negative effect of grain sizes determined the corrosion resistance of the sample, so the corrosion rate increased accordingly. The results of immersion and electrochemical tests were incompletely identical due to the different corrosion rates in the long-term and short-term tests [33].

**Figure 7:**
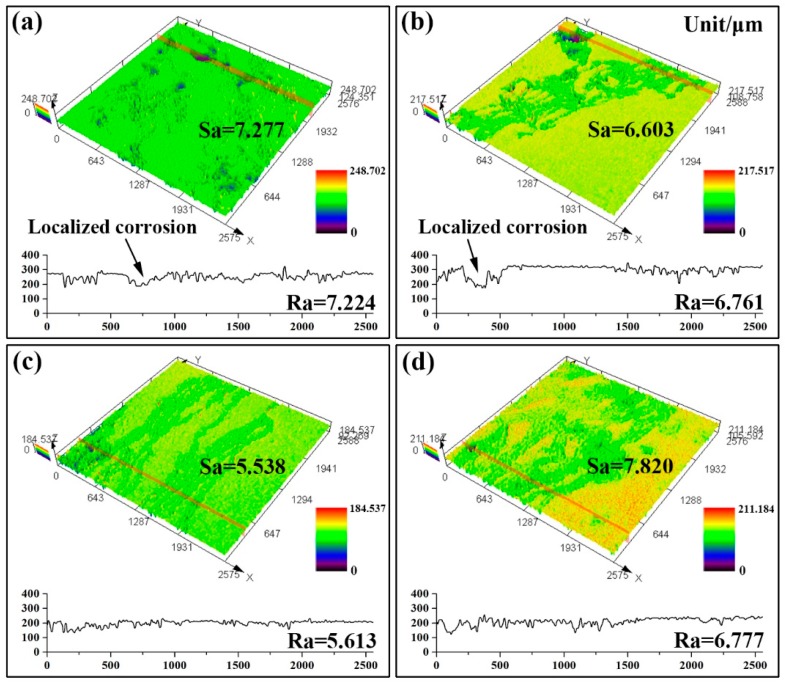
The three-dimensional (3D) corrosion morphologies of the samples after removing corrosion products: (**a**) as-cast; (**b**) 470 °C; (**c**) 490 °C; (**d**) 510 °C.

As shown in Figure 7, the three-dimensional (3D) corrosion features of the samples after removing corrosion products were observed by CLSM. As indicated, columns with different colors showed different corrosion morphologies, while the lowest area is blue and the highest area presents as red. The as-cast sample in Figure 7a was severely corroded with many pits. The T4-470 sample (Figure 7b) presents a few deep pits due to the incomplete solid solution and the uneven distribution of residual second phases. The corroded surface of the T4-490 sample was smooth without obvious localized corrosion. The depth difference (Ra) and surface roughness (Sa) both can reflect the corrosion resistance of the samples. The smaller values of Ra and Sa mean the better corrosion resistance. The Ra values of the as-cast, T4-470, T4-490 and T4-510 were 7.224, 6.761, 5.613 and 6.777 μm, respectively, which was roughly the same tendency with the results of weight loss corrosion rate and electrochemical test. The Sa values of the as-cast, T4-470, T4-490 and T4-510 are 7.277, 6.603, 5.538 and 7.820 μm, indicating that the corrosion resistance of the samples could be ranked in decreasing order as follows: T4-510 > as-cast > T4-470 > T4-490. However, in Figure 7a,b), the Ra values of as-cast and T4-470 samples crossing the localized corrosion region were larger than the Sa values. With the increasing of solution temperature to 490 °C, due to the decrease of the volume fraction of the second phase, the tendency of microgalvanic corrosion along the interface between the second phase and the matrix was reduced leading to the reduction of local corrosion. However, despite the better solid solution effect, the 3D corrosion morphology of 510 °C has higher Ra and Sa value and the sample was continually corroded due to the bulky and uneven grain size. The abnormal growth of grain boundary could not effectively prevent the occurrence of corrosion, so the corrosion is uniformly diffused. Among all the samples, the T4-490 sample, presented a lower roughness than the other samples.

**Figure 8:**
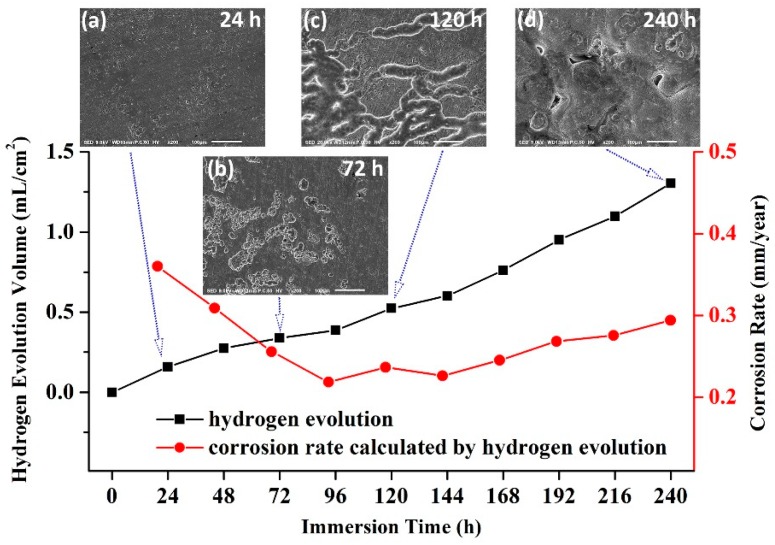
Extent of corrosion for T4-490 sample in SBF as a function of immersion time: (**a**) 24 h; (**b**) 72 h; (**c**) 120 h; (**d**) 240 h.

To further confirm the corrosion form of T4-490 sample, the extent of corrosion in SBF as a function of immersion time is presented in Figure 8. The accumulation of hydrogen by immersing the sample in SBF was calculated. For a short time (24 h), the surface of the sample was flat and had a small shallow corrosion pit. The corrosion initiates at the residual second phase, but the second phase peeled during cleaning the corrosion products [34]. In the range of 24 h to 72 h, the small corrosion points continued to corrode along the residual second phase in terms of depth and width. With the extension of immersing time to 120 h, the corrosion rate calculated from hydrogen evolution descended to the stable value. The number of filiform corrosion from the enlarged corrosion pits gradually increased and the corrosion area continued to expand at 120 h. In the absence of a new remaining phase, corrosion developed laterally. Thus, the corrosion mechanism was relatively uniform corrosion for the T4-490 sample.

## 4. Conclusions

(1) The as-cast Mg-Zn-Gd-Y-Zr alloy is mainly composed of α-Mg, lamellar structure Mg_12_Zn(Gd,Y) phase, skeleton-like Mg_3_Zn_3_(Gd,Y)_2_ phase and cubic-like (Mg,Zn)_3_(Gd,Y) phase. At the range of 470 °C~510 °C, Mg_12_Zn(Gd,Y) phase completely dissolves with a small amount of residual similar ellipse phase left. The volume fraction of second phase decreases significantly from 3.07% ± 0.37% of as-cast alloy to 0.70% ± 0.23% of T4-470, 0.34% ± 0.13% of T4-490 and 0.11% ± 0.03% of T4-510.
(2) After solution treatment, with the increase of the matrix potential and the decrease of the residual second phase, the sample shows better corrosion resistance. The remaining phase of solution-treated at 490 °C for 8 h exhibits relatively homogeneous dissolution. The corrosion rate and surface roughness are 0.472 ± 0.048 mm/year and 5.538 μm, respectively.
(3) For all the investigated samples, the T4-490 sample exhibits much better corrosion resistance and more uniform corrosion characteristic. With the extension of immersing time to 240 h, corrosion rate tends to be stable with a more uniform corrosion morphology.

## Figures and Tables

**Table 1:** The grain size and second phase volume fraction of the samples.

Samples	As-cast	T4-470	T4-490	T4-510
Grain size (μm)	68.1 ± 6.6	79.0 ± 1.4	95.1 ± 3.8	112.5 ± 10.5
Volume fraction of second phase (%)	3.07 ± 0.37	0.70 ± 0.23	0.34 ± 0.13	0.11 ± 0.03

**Table 2:** Electrochemical parameters of the samples obtained from the polarization curves.

Samples	E_corr_ (V)	E_b_ (V)	−βc (V/dec)	I_corr_ (μA/cm^2^)	P_i_ (mm/year)
As-cast	−1.571	−1.514	0.167	4.688	0.107
T4-470	−1.504	−1.423	0.160	4.338	0.099
T4-490	−1.457	−1.361	0.149	3.102	0.071
T4-510	−1.522	−1.472	0.169	5.334	0.122
